# Supplementary figures and images for: Non-destructive DNA extraction for recovering mitochondrial genomes from museum grasshopper specimens
Source: PLoS One. 2026 Feb 2;21(2):e0341621. doi: 10.1371/journal.pone.0341621 (PMC12863550; doi:10.1371/journal.pone.0341621)

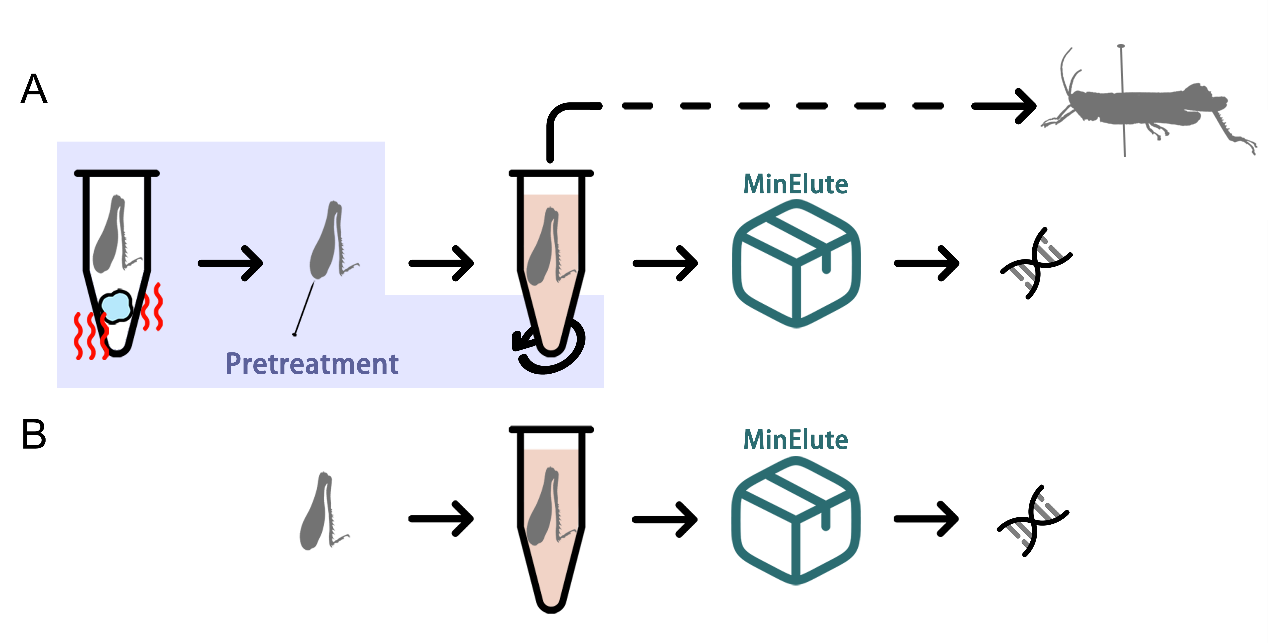

Supplement: S1 Fig — (TIF) [file pone.0341621.s001.tif]

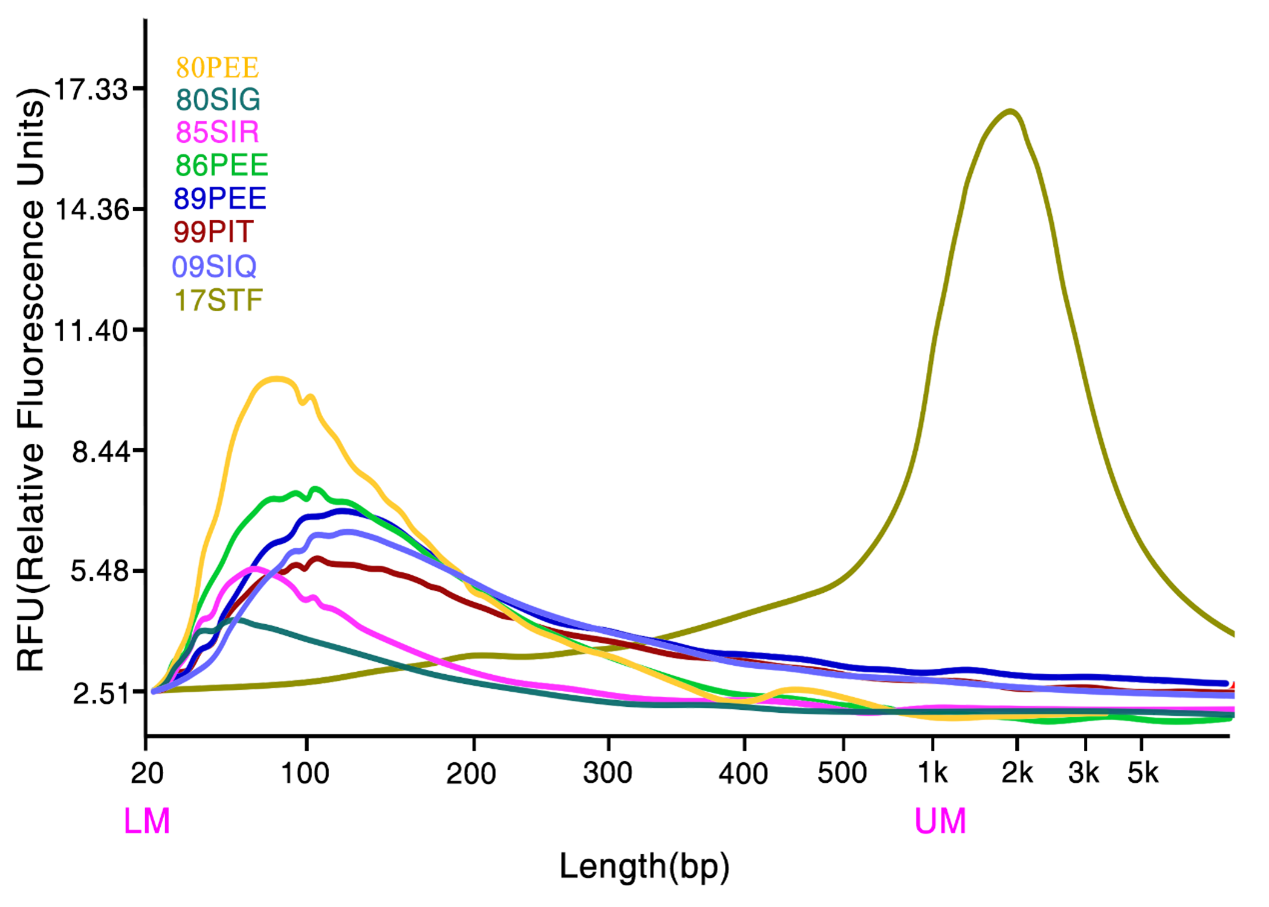

Supplement: S2 Fig — The DNA of different specimen was distinguished by color. LM and UM represent lower (20 bp) and upper marker (1 kbp), respectively. (TIF) [file pone.0341621.s002.tif]

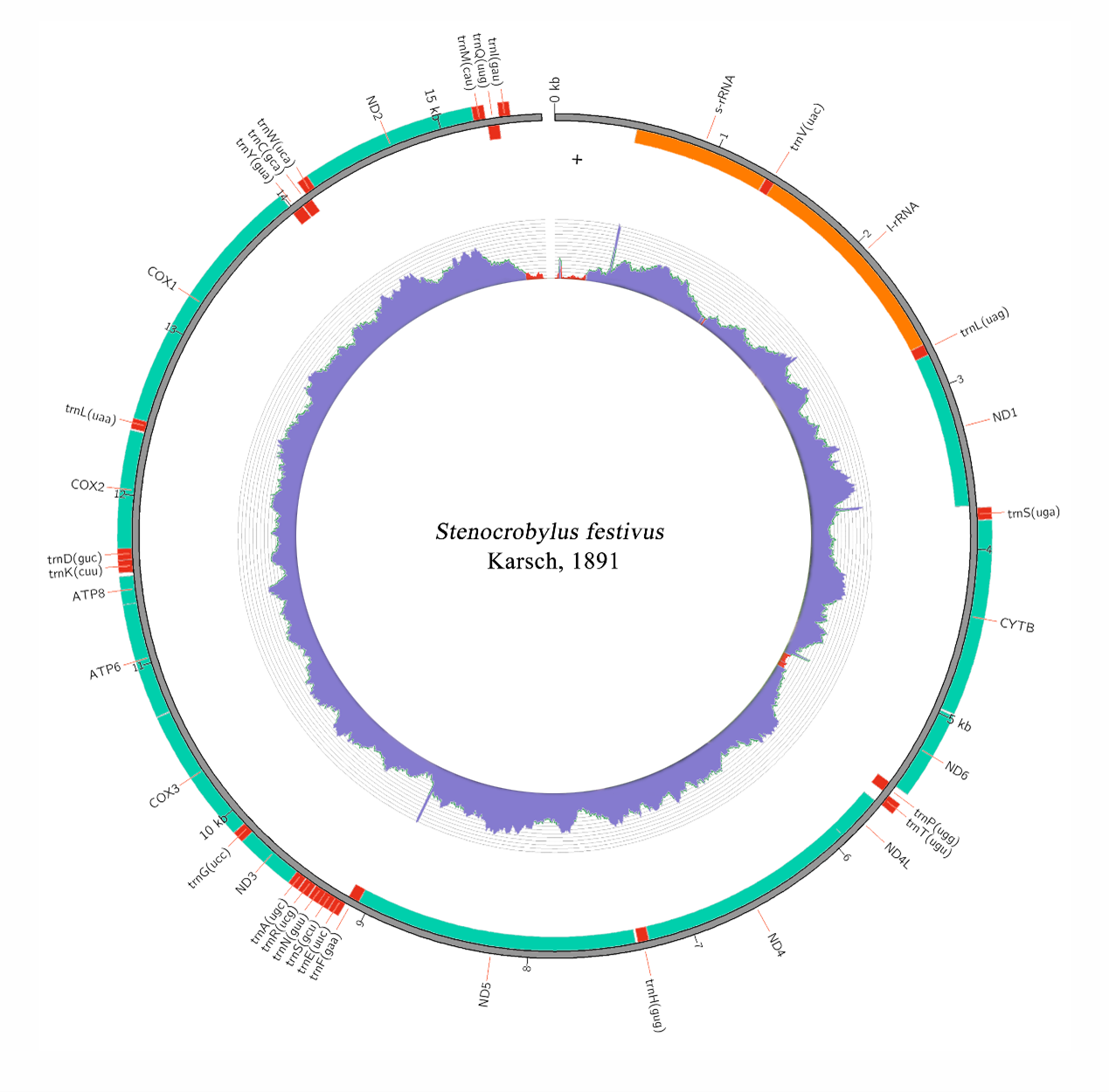

Supplement: S3 Fig — Cyan, red and orange represent protein-coding, tRNA and rRNA genes, respectively. Inner purple circle represents assembly depth. (TIF) [file pone.0341621.s003.tif]

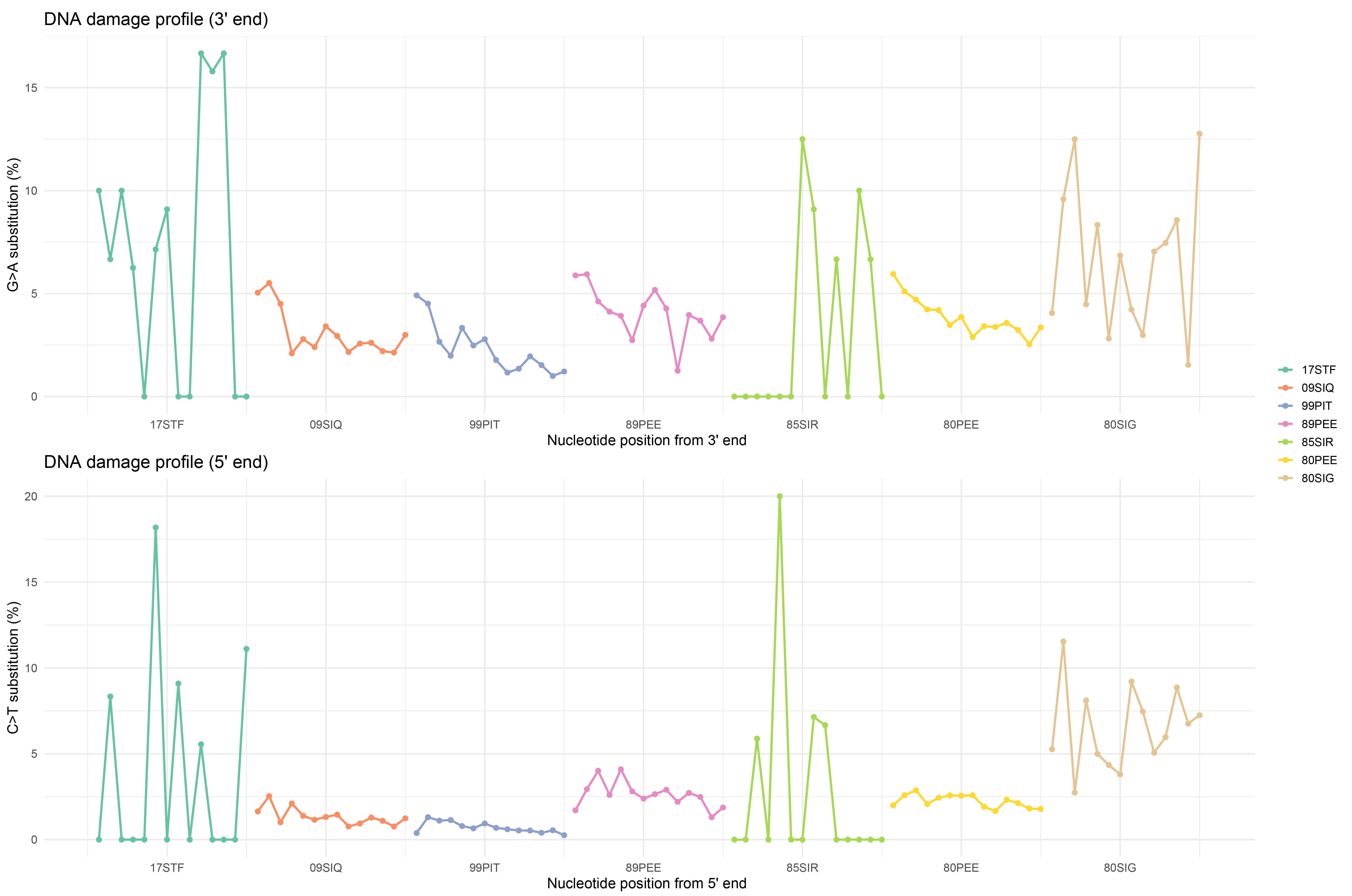

Supplement: S5 Fig — (TIF) [file pone.0341621.s005.tif]

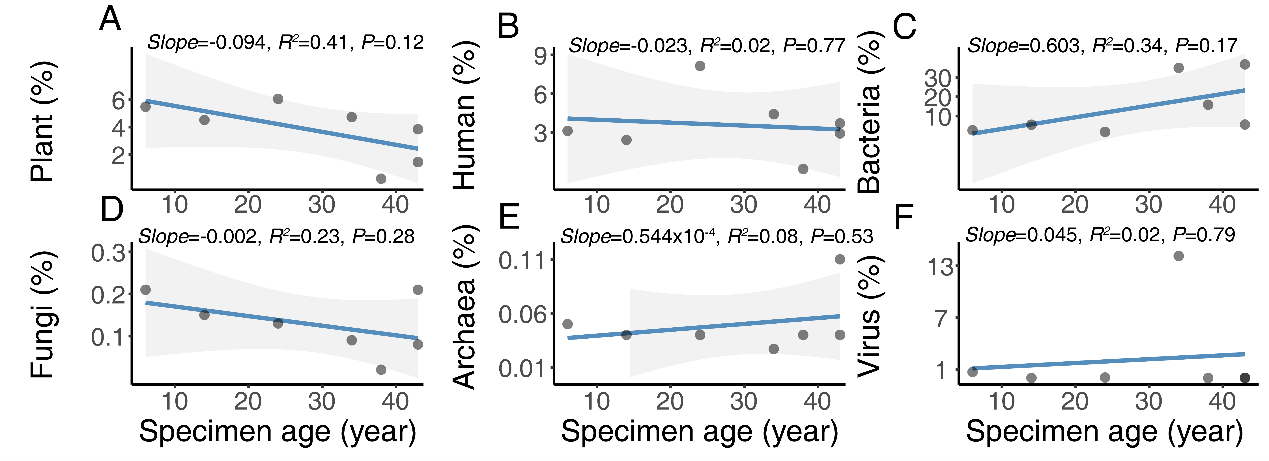

Supplement: S6 Fig — Linear regression lines, 95% confidence interval (shaded areas) and associated statistics are displayed. (TIF) [file pone.0341621.s006.tif]
