# Supplementary material for: Non-destructive DNA extraction for recovering mitochondrial genomes from museum grasshopper specimens
Source: PLoS One. 2026 Feb 2;21(2):e0341621. doi: 10.1371/journal.pone.0341621 (PMC12863550; doi:10.1371/journal.pone.0341621)
Supplement: S1 File — The full protocol is deposited at protocol.io (DOI: dx.doi.org/10.17504/protocols.io.bp2l6zj95gqe/v1). (DOCX) [file pone.0341621.s009.docx]

**Supporting file: DNA extraction protocol for dried grasshopper specimens**

**For article:**

**Mitochondrial Genomes from Museum Grasshopper Specimens via Non-Destructive DNA Extraction**

Hao Tang^1¶^, Jiayi Zou^1¶^, Keyao Zhang^1^, Huateng Huang^1,*^

. College of Life Science, Shaanxi Normal University, Xi’an, 710000, China

*Corresponding author:

E-mail: huanghuateng@snnu.edu.cn (SNNU)

^¶^These authors contributed equally to this work.

**Material**

MinElute PCR Purification Kit (Qiagen Catalog #28004)；

Tris (Biotopped, T6061);

NaCl (BKMAMLAB, 10019318);

EDTA (Tianjin Kemiou Chemical Reagent, #20170407);

SDS (Beyotime, ST627);

HCl, 1N (Codow, CD433036);

Protease K, 20 mg/ml (Beyotime, ST532);

Tween 20, 100% (G-CLONE, CS9028);

Sodium acetate, 3M (Sigma-Aldrich, #126-96-5);

Buffer PB (Qiagen, #19066);

Buffer PE (Qiagen, #19065);

**Preparation**

1. **Buffer Preparation (Binding Buffer)**
   To prepare 100 mL of sterile pH 8 binding buffer (200 mM Tris, 250 mM NaCl, 25 mM EDTA, 0.5% SDS):
   - Weigh 2.422 g of Tris, 1.461 g of NaCl, 0.73 g of EDTA, and 0.5 g of SDS.
   - Add the solids to a clean beaker with 80 mL of sterile water.
   - Stir the mixture on a magnetic stirrer (SilentShake, #MS-01H) at 56 °C, 850 rpm, for 2 hours until fully dissolved.
   - Transfer the solution to a clean blue-cap bottle and autoclave it for sterilization.
   - After cooling, adjust the pH to 8 with HCl.
   - Carefully transfer the solution to a volumetric flask and adjust the volume to 100 mL with sterile water.
   - Store the final buffer in a sterile blue-cap bottle.
2. **Lysis Buffer A (with Proteinase K)**
   To prepare 5 mL of Lysis Buffer A:
   - Mix 4.9 mL of binding buffer with 0.1 mL of 20 mg/mL Proteinase K in a 15-mL centrifuge tube.
   - Invert the tube gently to mix thoroughly.
3. **TET Buffer Preparation**
   To prepare 100 mL of pH 8 TET buffer (10 mM Tris, 1 mM EDTA, 0.05% Tween-20):
   - Weigh 0.121 g of Tris and 0.029 g of EDTA, and dissolve them in 80 mL of sterile water in a clean beaker.
   - Stir the mixture on a magnetic stirrer (SilentShake, #MS-01H) at 56 °C, 900 rpm, for 1–2 hours.
   - Transfer the solution to a sterile blue-cap bottle and autoclave.
   - Once cooled, add 50 µL of 100% Tween-20, and stir at 56 °C, 900 rpm, for 30 minutes.
   - Adjust the pH to 8 using HCl.
   - Transfer to a volumetric flask and adjust the final volume to 100 mL with sterile water.
   - Store the buffer in a sterile blue-cap bottle.

**Sample Washing and Rehydration**

1. Place grasshopper hind legs on clean filter paper.
2. Using sterile tweezers, hold the tibia and rinse the hind legs with 1 mL of absolute ethanol using a pipette.
3. Allow the rinsed legs to air dry on clean filter paper.
4. Add a small piece of moistened sterile filter paper into a 2-mL centrifuge tube.
5. Place the dried hind legs into the tube and incubate at 37 °C in a metal bath (OHAUS, HB2DGHL) for 3 hours to rehydrate.

**Sample Digestion**

1. Remove the hind legs from the rehydration solution and air dry on filter paper.
2. Using sterile insect needles, pierce the femur from the trochanter-femur joint and transfer the leg to a clean centrifuge tube.
3. Add 200 µL of Lysis Buffer A to the tube, ensuring the tissue is fully submerged.
   *Note: Increase the volume of Lysis Buffer A until whole tissue soaked.*
4. Place the tube in a thermomixer (JingXin, #JXH-200) and incubate at 56 °C, 400 rpm, overnight.

**DNA Extraction with MinElute Kit**

1. Prepare a binding solution by mixing 55 µL of 3 M sodium acetate and 1,400 µL of Buffer PB in a 2-mL centrifuge tube.
2. Transfer 200 µL of lysate to the binding solution and mix gently.
3. Transfer 450 µL of the mixture to a MinElute spin column and centrifuge at 3,600 rpm for 4 minutes. Discard the flow-through.
4. Repeat step 3 until all lysate-binding mixture is processed (typically 3 rounds).
5. Dry-spin the column at 5,400 rpm for 1 minute to remove residual liquid.
6. Wash the column with 450 µL of Buffer PE and centrifuge at 5,400 rpm for 1 minute. Discard the flow-through.
7. Repeat the wash step once more.
8. Perform a final dry spin at 13,000 rpm for 1 minute, then transfer the column to a clean 1.5-mL centrifuge tube.
9. Elute DNA by adding 25 µL of Buffer TET to the silica membrane, incubating at room temperature for 5–10 minutes, and centrifuging at 7,300 rpm for 1 minute.
10. Repeat the elution step, collecting a total of 50 µL of DNA.

**Supplementary figures**

Fig. S1 Diagrams of DNA extraction workflow, showing the difference between lysis with the pretreatment (A) and minimal-disturbance setting (B).

Fig. S2 Fragment sizes of extracted DNA from dried grasshopper specimens. The DNA of different specimen was distinguished by color. LM and UM represent lower (20bp) and upper marker (1 kbp), respectively.

Fig. S3 The assembled mitochondrial genome of *St. festivus*. Cyan, red and orange represent protein-coding, tRNA and rRNA genes, respectively. Inner purple circle represent assembly depth.

Fig. S4 DNA damage, represented by deviations in C to T substitutions frequency at the 5'-ends’ 1^st^ (A), 2^nd^ (B), and 3^rd^ (C) position relative to the average of 5^th^-15^th^ position. Correlations between the frequency deviations in G to A substitutions at the 3'-ends of the reads — 1^st^ (D), 2^nd^ (E), and 3^rd^ (F) position—with specimen age. Linear regression lines, 95% confidence interval (shaded areas) and associated statistics are displayed.

Fig. S5 Relationship between sample age and others exogenous DNA. Linear regression lines, 95% confidence interval (shaded areas) and associated statistics are displayed.
