# Supplementary material for: Non-destructive DNA extraction for recovering mitochondrial genomes from museum grasshopper specimens
Source: PLoS One. 2026 Feb 2;21(2):e0341621. doi: 10.1371/journal.pone.0341621 (PMC12863550; doi:10.1371/journal.pone.0341621)
Supplement: Attachment — Submitted filename: Response to Reviewers.docx [file pone.0341621.s011.docx]

**Reviewer #1:**

The study lacks a rigorous assessment of endogenous DNA content. Although exogenous contamination is quantified using Kraken2, the proportion of authentic grasshopper DNA in the extracts remains unverified. Given the high variability in contamination levels (50% in some specimens), this raises concerns about the protocol’s efficiency in retrieving target DNA. Incorporating negative controls during extraction and quantifying endogenous DNA would provide critical validation.

Response:

We thank the reviewer for this valuable suggestion. We now **include the proportion of authentic grasshopper DNA in the Sankey plots** (Fig. 2), providing a direct quantification of endogenous DNA content. Besides, it is important to note that variable contamination levels are a common feature in historical DNA studies, often reflecting specimen storage conditions rather than extraction performance. Despite some samples showing high exogenous fractions, our protocol consistently yielded sufficient endogenous DNA to assemble complete mitochondrial CDS. This demonstrates that the method is effective for recovering authentic target sequences, even under less-than-ideal preservation scenarios. We did add a negative control and quantified its dsDNA amount using Nanodrop, but didn’t sequence it. We **add it in our table 1**.

The title suggests a focus on mitochondrial genomes, yet the paper does not leverage these data for evolutionary or ecological insights. While the assembly of *Stenocrobylus festivus* mitogenomes is a valuable proof of concept, the opportunity to explore phylogenetic relationships, temporal genetic shifts, or comparative analyses with modern specimens is missed. To align the manuscript’s scope with its title, the authors should either reframe the title to emphasize methodology or include preliminary evolutionary analyses.

Response:

We agree with the reviewer’s assessment. To better reflect the methodological focus of the study, we have changed the title to:“Non-Destructive DNA Extraction for Recovering Mitochondrial Genomes from Museum Grasshopper Specimens.”This revised title avoids overstatement of evolutionary analyses while clearly emphasizing the protocol and its application.

**Reviewer #2:**

1) Since the manuscript is intended as a methodological paper, the experimental procedures, including design, sampling, library preparation, and data analysis, should be described in much greater detail to allow proper assessment and reproducibility. As it stands, the methodological sections, in particular library preparation or data analysis, are too sparse to support a clear methodological advancement.

Response:

We have expanded the methodological sections to provide greater detail on the library preparation procedures and contamination analyses. This now allows for more transparent assessment and reproducibility. Besides, a clear pipeline for DNA sampling is listed in supplemental file 1.

2) I also suggest moving the table S1 to the main text.

Response:

We have moved the former Supplementary Table S1 into the main text as Table 1.

3) The diagram of the DNA extraction workflow (Figure S1) is well designed and highly informative. However, I recommend that the authors ensure full consistency between Figure S1, the main text, and Supplementary Table S1 with respect to the naming or labeling of the extraction methods applied to each sample. Currently, there appear to be some ambiguities in how the methodologies are referred to across different parts of the manuscript and supplementary materials, which could make it difficult for readers to follow or reproduce the experimental design.

Response:

We carefully revised the manuscript to ensure consistent terminology across Figure S1, the main text, and the tables. We delete those unclear characters.

4) This sentence can be better explained or reformulated: l. 84 “Due to the size constraints of a 1.5 ml centrifuge tube, the hind leg of each grasshopper specimen was initially removed”. It is not clear if the leg was totally removed from the tube, or when it was placed back in the tube.

Response:

We revised the sentence to clarify the step.

5) Statistical support: some of the reported improvements are not statistically significant (e.g., P = 0.06), and yet are described in the text as if they were. Moreover, the statistical analyses employed should be more clearly described, and the authors should avoid overinterpreting marginal or non-significant results.

Response:

We deleted the term “significantly” where the P-value was marginal (e.g., P = 0.06) and revised the statistical descriptions to avoid overinterpretation.

6) Inappropriate or incomplete analyses: the bioinformatic approach appears suboptimal or not fully adapted to the nature of the degraded DNA typically recovered from museum specimens and probably also to the chemistry of the sequencing platform. A useful pipeline could be represented by nf-core/eager (Fellows Yates et al., 2021). More justification for the chosen tools and pipelines, as well as a discussion of potential limitations, is needed. For example, the authors report the presence of polyG-rich reads or sequences. However, it is unclear whether these observations reflect technical artifacts arising from either pipeline limitations or the sequencing chemistry due to 2-channel chemistry or read length (with PE150 sequencer may read into the adapter or generate lowquality tail regions, where polyG miscalls are frequent — especially in the absence of signal toward the end of the read).

Response:

We have incorporated the nf-core/eager pipeline to process our data, added corresponding method and results. We compare our previous DNA damage result, and revise its with eager’s output. For poly-G, we completely understand its relation to the length of DNA insertion, thus we change its expression in the *Sequence analyses* section.

7) More about bioinformatic approach: a method to improve mappings on circular genomes is CircularMapper. Moreover, more details about the parameters selected for reconstructing the consensus sequences need to be provided.

Response:

We tested the CircularMapper (CM) tool by installing it via Conda. Specifically, we applied CM to sample 99PIT and 17STF and compared the BAM results with our previous mapping approach. Here’s our main steps to generate the BAM via CM:

*$java -jar /home/DataDisk/th_data/software/envs/CM/share/circularmapper-1.93.5-3/generator-1.93.5.jar -e 500 -i pi.zhengi.fasta -s "chrMT"*

*$bwa aln -t 4 pi.zhengi_500.fasta your.merged.fastqfile -n 0.04 -l 32 -f output.sai*

*$bwa samse pi.zhengi_500.fasta output.sai 99BTM_merge.fastq -f output_circmapper.sam*

*$java -jar /home/DataDisk/th_data/software/envs/CM/share/circularmapper-1.93.5-3/realign-1.93.5.jar -e 500 -i output_circmapper.sam -r pi.zhengi.fasta*

We generate consensus sequence by Geneious prime, and map them to the reference. Although we found that in 99PIT, SNPs are fewer using CM, but appears a 196 bps gap in the sequence, and for 17STF, we find very few reads successfully mapped to the reference in 17STF using CM (We think it might cause by remoted relation between *St. festivus* and its reference *Diabolocatantops_pinguis*, for which is the only available reference of the same family). Although CM calls BWA-aln module, our sequencing strategy produce 150 bp pair end reads from historical DNA, and according to Dolenz’s research (Dolenz, 2024) BWA-mem still perform better toward 150 bp. So, we retained our original results, but further add a comment of CircularMapper.

8) Low sample size and lack of controls: the most critical limitation of the study is the relatively small and uneven sample size, especially in relation to the number of species, lysis buffers, and incubation times tested. The low number of replicates per condition significantly limits the statistical power of the analyses and the generalizability of the findings. I strongly encourage the authors to explicitly acknowledge this limitation in the manuscript and to refrain from overinterpreting marginal or non-significant results. If possible, the inclusion of additional replicates would considerably strengthen the study’s conclusions.

Response:

We agree and now explicitly acknowledge the limitation of sample size and replication in the revised Discussion.

9) Lack of negative controls (e.g., extraction blanks): they are not mentioned in the text. They usually can assess potential contamination when working with historical material.

10) In the discussion the authors stated that “Our protocol yields 1,750–13,495 ng of DNA per specimen, surpassing values reported for grinding-based protocols (e.g., 18.31–325 ng)”. I would strongly recommend removing or rephrasing this comparison, as it lacks sufficient contextual basis. The cited values come from a different study involving samples of unclear geographic origin, age, storage conditions, preservation history, or library preparation method, which may differ substantially from those used in the present study. If the authors wish to reference previously reported yields, it would be more appropriate to do so in a descriptive or contextual manner, without implying a direct performance comparison.

Response:

We did include a negative control. Although we did not sequence it, we quantified its dsDNA amount using Nanodrop. We now report this information in the revised Table 1 and discuss it in the result. Trace DNA yield in negative control shows limited contamination introduction.

11) It appears that only one mitochondrial genome has been deposited in GenBank. Could the authors clarify why the remaining mitogenomes were not submitted, and whether there are plans to make them publicly available?

Response:

No species of the genus *Stenocrobylus* had any sequences deposited in GenBank prior to our study; thus, we report it here for the first time. For the other species successfully assembled through consensus generation, corresponding sequences are already available in NCBI. We attempted to submit these as well, but received responses such as ‘Some or all of the sequences contain little similarity to other sequences in the database based on BLAST searches.’ Therefore, we now mark them as unverified, and they will be released to the public database upon publication.
