# Supplementary material for: Non-destructive DNA extraction for recovering mitochondrial genomes from museum grasshopper specimens
Source: PLoS One. 2026 Feb 2;21(2):e0341621. doi: 10.1371/journal.pone.0341621 (PMC12863550; doi:10.1371/journal.pone.0341621)
Supplement: Attachment — Submitted filename: Response_to_Reviewers_auresp_2.docx [file pone.0341621.s012.docx]

**Reviewer #2:**

First, I recommend expanding the discussion of deamination patterns, also in light of the findings reported by Korlević et al. (2021) and Modi et al. (2021). Integrating these studies would provide useful context and help clarify how your results fit within the broader literature on ancient/historical DNA damage.

Response:

We have expanded the Discussion to better describe DNA damage patterns observed in our study. We note that assessing these damage patterns helps distinguish endogenous DNA from contamination and informs mapping and assembly strategies. The manuscript has been revised accordingly.

Second, I would like to comment on the statement:

“Although CM calls the BWA-aln module, our sequencing strategy produced 150 bp paired-end reads from historical DNA, and according to Dolenz’s research (Dolenz, 2024) BWA-MEM still performs better for 150 bp. So, we retained our original results, but further added a comment on CircularMapper.”

I do not fully agree with this reasoning. Your reads are indeed 150 bp as raw output from the sequencer; however, in practice they are very likely much shorter once adapter trimming is applied. Given the short insert sizes typical of historical DNA, the opposite adapter will often be reached again, producing artificially long reads with terminal poly-G stretches. After trimming, the reads used for mapping will therefore be substantially shorter than 150 bp. For this reason, BWA-MEM may not be the most appropriate choice in this context. I suggest clarifying this potential scenario in the manuscript and discussing its implications for the mapping strategy.

Response:

We agree that, despite the nominal 150 bp read length, historical DNA libraries often produce substantially shorter trimmed reads because short inserts frequently reach the opposite adapter and generate poly-G tails. This scenario may indeed reduce the suitability of BWA-MEM, which is optimized for longer reads. In response to the reviewer’s suggestion, we have added a paragraph in the Discussion acknowledging this issue and clarifying that short trimmed reads may be better handled by algorithms tailored to degraded DNA, such as BWA-aln or the internal mapping module used by CircularMapper. We also note this as an important consideration for future applications of our pipeline. The manuscript has been revised accordingly.
